# Human epidermal growth factor receptor 3 serves as a novel therapeutic target for acral melanoma

**DOI:** 10.1038/s41420-023-01358-5

**Published:** 2023-02-10

**Authors:** Yuka Tanaka, Takamichi Ito, Yumiko Kaku-Ito, Keiko Tanegashima, Gaku Tsuji, Makiko Kido-Nakahara, Yoshinao Oda, Takeshi Nakahara

**Affiliations:** 1 Department of Dermatology, Graduate School of Medical Sciences, Kyushu University, Fukuoka, 812-8582 Japan; 2 Department of Anatomic Pathology, Graduate School of Medical Sciences, Kyushu University, Fukuoka, 812-8582 Japan

**Keywords:** Melanoma, Skin cancer

## Abstract

Acral melanoma (AM) is a rare, life-threatening skin cancer. Since AM bears unique features, existing therapies for other types of malignant melanomas have limited effects and the establishment of effective treatments for AM is strongly desired. Human epidermal growth factor receptor 3 (HER3) is a receptor tyrosine kinase that is frequently elevated in tumors and contributes to tumor progression, so it is considered a promising therapeutic target for tumors. This study was established to evaluate the potential of HER3-targeted therapy to treat AM by investigating the expression and function of HER3. HER3 expression was immunohistochemically analyzed in AM lesions of 72 patients and in AM cell lines. To investigate function of HER3, effects of HER3 inhibition on cell proliferation, apoptosis/survival, anchorage-independent growth, and underlying signals were assessed. HER3 was expressed in patients’ AM tissues with various intensities and HER3 expression was significantly correlated with patient’s disease-free survival. In vitro analyses revealed that HER3 is more highly expressed in AM cells than in normal epidermal melanocytes. AM cells were also shown to be sensitive to the cytotoxic part of a HER3-targeted antibody-drug conjugate. Inhibition of HER3 did not affect cell proliferation, whereas it decreased the anchorage-independent growth of AM cells likely through affecting the nuclear translocation of Yes-associated protein. It is implied that HER3 may serve as a novel therapeutic target for AM.

## Introduction

Malignant melanoma is one of the most common lethal skin tumors, originating from melanocytes. Acral melanoma (AM) is a rare subtype of cutaneous melanoma, with a distinct genetic background. AM is found in acral locations such as the palms, soles, fingers, toes, and nail apparatus, usually accompanied by lentiginous atypical melanocytic spread in the epidermal basal layer [1, 2]. Thus, histopathologically, most AM cases are acral lentiginous melanoma. The frequency of AM varies depending on ethnicity, accounting for 33–35%, 18–23%, 9%, and 1% of cutaneous melanoma cases in Blacks, Asians/Pacific Islanders, Hispanic Whites, and non-Hispanic Whites, respectively [3], although the other subtypes are more common in Whites. Mechanical stress or a history of trauma is thought to be risk factors for AM. Unlike the other types of melanoma, ultraviolet radiation is not related to the occurrence of AM [2, 4, 5]. AM shows a poorer prognosis than other subtypes, with a 5-year survival rate of about 60–80% and a 10-year survival rate of about 40–60% [5–7].

AM is treated by surgical resection when found at an early stage. Unresectable, advanced, or metastasized AM is treated by combined approaches including radiation therapy, classical chemotherapy, immune checkpoint inhibitors, and targeted therapies [8]. However, because of its low incidence and unique characteristics, effective treatments for AM have not been established [9–11]. Currently, BRAF inhibitors in combination with MEK inhibitors are applied for treating melanoma since about 50% of non-acral melanomas possess mutated BRAF (mainly BRAF^V600E^), which causes aberrant activation of the downstream mitogen-activated protein kinase (MAPK) signals, leading to the proliferation of cancer cells [12–17]. However, BRAF mutation is not common in AM [18]. Instead, mutation/amplification of *KIT*, mutation of *Cyclin D1* (*CCND1*), and amplification of *GAB2* are more frequently found in AM [19–23]. KIT proto-oncogene is a receptor tyrosine kinase and about 20–30% of AM reportedly bears the *KIT* mutations which locate in exons 11, 13, 17, or 18. The observed mutations were, Y553N, R634W, K642E (an oncogenic mutation), and so on. In addition, a narrow amplification on 4q12 was found [18, 20]. GAB2 is a scaffold protein which mediates various signaling pathways. *GAB2* amplification located on 11q14.1 and there was a limited number of cases that *GAB2* amplification exists with *KIT* amplification [22]. These mutations/amplifications may serve as therapeutic targets for AM, but the efficacy of such approaches is still unclear [9, 20].

Human epidermal growth factor receptor 3 [HER3, also known as Erb-B2 receptor tyrosine kinase 3 (ErbB3)] is a member of the HER receptor tyrosine kinase family. This family consists of four highly homologous members, HER1–4, which play roles in normal and tumor cell biology [24, 25]. HER3 mainly dimerizes with HER1/2, which in turn binds to ligands such as neuregulins (NRGs) to activate the downstream signaling pathways phosphoinositide-3-kinase (PI3K)/protein kinase B (Akt) and MAPK, regulating cell proliferation and survival [24–26]. HER3 is reportedly highly expressed in melanoma compared with the levels in normal skin and other skin cancers [27, 28]. Low expression of HER1/2 and high expression of HER3 were reported to be detrimental to the 5-year survival of melanoma patients [29]. It has also been suggested that neuregulin 1 (NRG1)/HER3 signaling may contribute to melanoma progression and metastasis [30]. Given this background, HER3 is considered to be a novel therapeutic target for melanoma. Antibody-drug conjugates (ADCs) are a novel type of targeted drug [31]. The HER3-targeted ADC patritumab deruxtecan has recently been developed and tested in several cancers such as EGFR-mutated non-small-cell lung cancer and breast cancer [32, 33]. Thus, it is expected that melanoma may also be treatable by HER3-targeted ADC. However, the potential of HER3 targeting to treat AM remains unclear because previous investigations mainly focused on non-acral melanomas.

The purpose of this study was to evaluate the potential of HER3 as a novel therapeutic target for AM. As such, HER3 expression was investigated in the AM tissues of patients and in the AM cell lines SM2-1 and MMG-1. HER3 was expressed in AM tissues and its expression level was correlated with patient survival. HER3 was also expressed in AM cell lines and in vitro functional assays revealed its involvement in regulating anchorage-independent growth of AM cells partly through the NRG1/HER3/Yes-associated protein (YAP) axis. Our results demonstrate the expression and the role of HER3 in AM and suggest that HER3 is a promising therapeutic target for treating AM.

## Results

### HER3 is expressed in AM tissues and correlated with patient survival

**Fig. 1:**
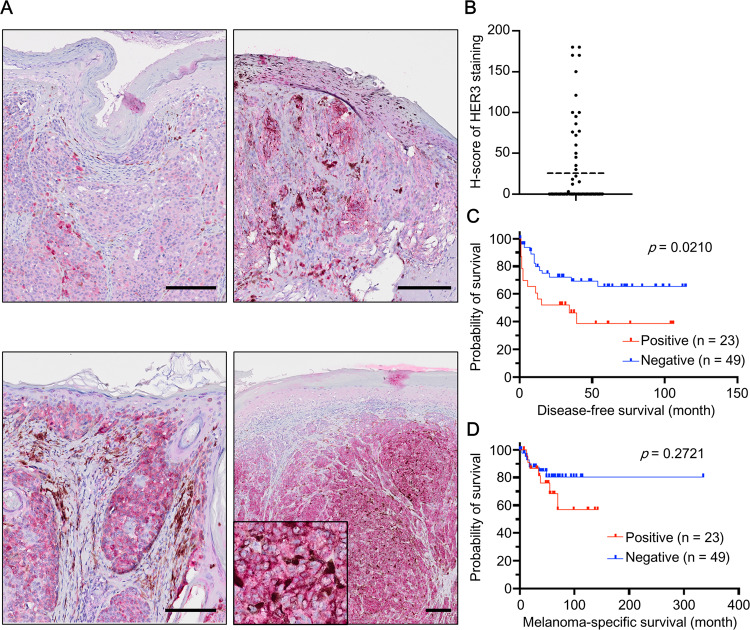
HER3 expression and relevance of HER3 intensity to the survival of AM patients. **A** AM tissues from 72 AM patients were immunohistochemically stained for HER3. Representative images are shown. Scale bars = 200 μm. **B** HER3 expression in clinical AM tissues was quantified using a semi-quantitative method and is shown as H-score. The H-score was obtained by multiplying the staining intensity (range 0–3+) by the percentage of HER3-positive cells. **C**, **D** AM patients were categorized into two groups: HER3-negative (*n* = 49) and HER3-positive (*n* = 23). Survival curves of (**C**) disease-free survival and (**D**) melanoma-specific survival are shown.

To investigate HER3 expression in AM, immunohistochemical staining was performed. Among 72 AM patients, 34 were male (47.2%) and 38 were female (52.8%), and the mean age was 65.4 (range 32–88). The primary site of AM was foot (62.5%), hand (13.9%), or nail apparatus (23.6%). HER3 was expressed in the AM lesions with various intensities (Fig. 1A). In some tissues, intra-tumor heterogeneity of HER3 intensity was observed. To further investigate the relevance of HER3 expression to patient survival, patients were categorized into HER3-positive (*n* = 23) and HER3-negative [*n* = 49; those with histological score (H-score) 0] groups based on the H-score of HER3 (Fig. 1B). The disease-free survival (DFS) and melanoma-specific survival (MSS) were then compared between HER3-negative and HER3-positive groups. MSS and DFS were calculated from the date of the first histopathological examination to the date of death as a result of melanoma or the date of any recurrence (local recurrence and metastasis). Data on patients without death or recurrence were censored on the date of the last follow-up before July 31, 2022, and data on patients who died of other causes were censored at the time of death. DFS was significantly shorter in the HER3-positive group than in the HER3-negative one (Fig. 1C). MSS also tended to be shorter in the HER3-positive group, but the difference was not statistically significant (Fig. 1D).

Relationships between HER3 positivity and clinicopathological factors were also analyzed. As shown in Supplementary Table S1, there was no significant correlation between HER3 positivity and age, sex, or tumor site. On the other hand, there was a significant correlation between HER3 positivity and Breslow thickness; that is, the HER3-positive group had greater Breslow thickness than the HER3-negative group. Besides, the HER3-positive group tended to have a more advanced stage of cancer [American Joint Committee on Cancer (AJCC) stage], although this did not reach statistical significance. These results implied that HER3 positivity is related to the degree of primary tumor progression in AM.

### HER3 is highly expressed in human AM cell lines, SM2-1 and MMG-1

**Fig. 2:**
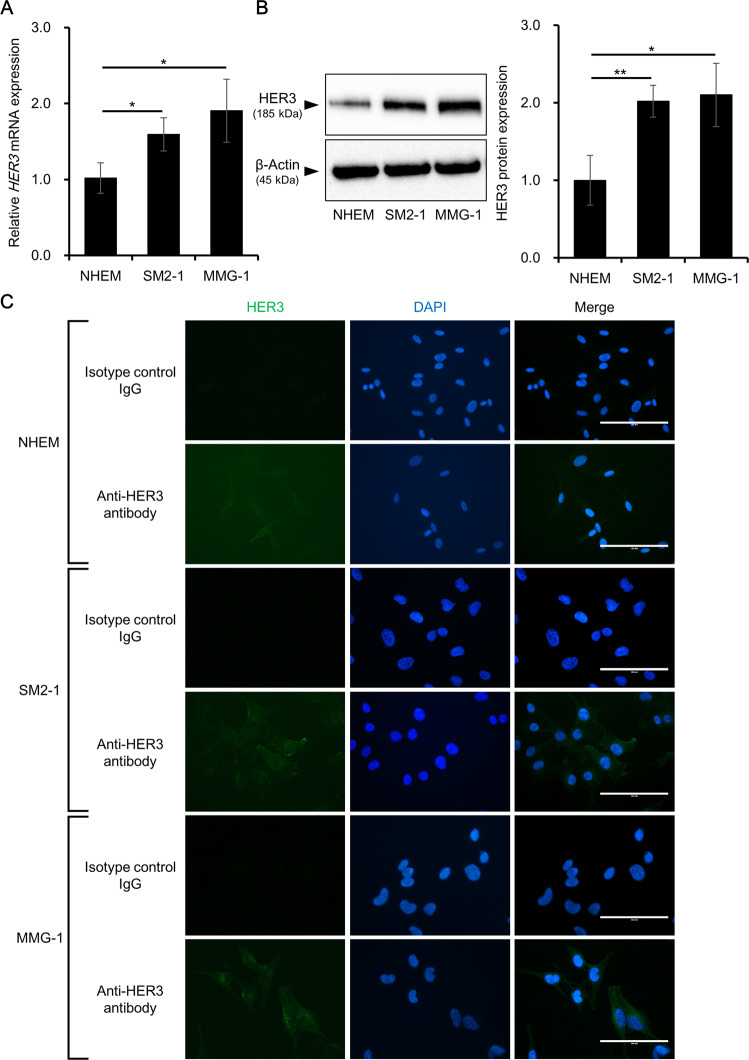
HER3 expression in normal and malignant melanocytes. HER3 expression was evaluated in normal melanocytes and in AM cells. **A** HER3 mRNA expression in normal human epidermal melanocytes (NHEM), SM2-1, and MMG-1 cells. Experiments were independently repeated three times and mean ± SD of HER3 mRNA expression is shown. *p < 0.05. **B** HER3 protein expression in NHEM, SM2-1, and MMG-1 cells. Experiments were independently repeated three times and representative blot images (left) and mean ± SD of HER3 protein expression (right) are shown. Original uncropped blot images are shown in Supplementary Fig. S1. *p < 0.05 and **p < 0.01. **C** Immunocytochemical staining of HER3 in NHEM, SM2-1, and MMG-1 cells. Experiments were independently repeated three times and representative images of HER3 (green, left panels), DAPI (blue, middle panels), and merged images (right panels) are shown. Scale bars = 100 μm.

We further analyzed HER3 expression in human AM cell lines. Normal human epidermal melanocytes (NHEM) were used to compare HER3 expression between normal and malignant melanocytes. HER3 was expressed at both mRNA and protein levels in NHEM, SM2-1, and MMG-1 cells (Fig. 2A, B and Supplementary Fig. S1). HER3 mRNA and protein expression was significantly higher in the AM cells than in NHEM. Immunocytochemical staining also confirmed that HER3 protein was expressed in these cells and the fluorescent signal of HER3 was significantly higher in AM cells than in NHEM (Fig. 2C and Supplementary Fig. S2).

### Knockdown of HER3 does not affect proliferation of AM cells

**Fig. 3:**
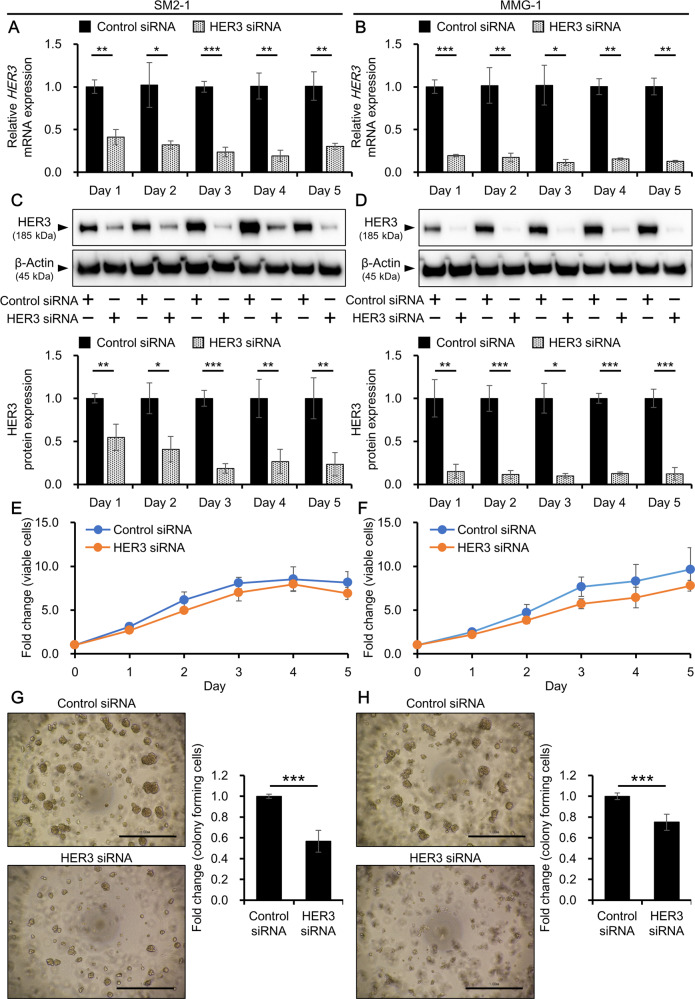
Effects of HER3 knockdown on proliferation and anchorage-independent growth of AM cells. AM cells were transfected with control or HER3 siRNA for 1–5 days and used for analyses. Knockdown efficiency of *HER3* mRNA in (**A**) SM2-1 and (**B**) MMG-1 cells. Experiments were independently repeated three times and mean ± SD of HER3 mRNA expression is shown. *p < 0.05, **p < 0.01, and *p < 0.001. Knockdown efficiency of HER3 protein in (**C**) SM2-1 and (**D**) MMG-1 cells. Experiments were independently repeated three times. Representative blot images (upper) and mean ± SD of HER3 protein expression (lower) are shown. Original uncropped blot images are shown in Supplementary Fig. S3. *p < 0.05, **p < 0.01, and *p < 0.001. Cell viability of siRNA-treated (**E**) SM2-1 and (**F**) MMG-1 cells was evaluated using a formazan-based assay. Mean ± SD of absorbance relative to that on day 0 is shown. Measurements were performed in five wells per condition and the experiments were independently repeated three times. **G**, **H** AM cells were transfected with control or HER3 siRNA, cultured in semisolid agar layers for 7 days, and evaluated for colony formation/anchorage-independent growth. Representative images and values of colony quantification by MTT assay in (**G**) SM2-1 and (**H**) MMG-1 cells. Scale bars = 1.0 mm. Experiments were independently repeated three times and mean ± SD of fold change of absorbance relative to the control siRNA-transfected condition is shown. ***p < 0.001.

To investigate the function of HER3 in AM, cells were transfected with control or HER3 siRNA and evaluated for their proliferation. The efficiency of HER3 knockdown was evaluated at mRNA and protein levels. In both cell lines, HER3 was significantly downregulated in HER3 siRNA-transfected cells compared with the level in control siRNA-transfected cells at the mRNA (Fig. 3A, B) and protein levels (Fig. 3C, D and Supplementary Fig. S3). Inhibition of HER3 slightly decreased the proliferation of AM cells, but the difference was not statistically significant (Fig. 3E, F). In accordance with this, the cell proliferation-related genes *CCND1* and *C-MYC* did not show distinct differences in expression between control siRNA-transfected cells and HER3 siRNA-transfected cells (Supplementary Fig. S4). To get some insights into apoptotic status of AM cells after HER3 knockdown, gene expressions of representative anti-apoptotic gene *BCL2* and pro-apoptotic gene *BAX* were evaluated [34, 35]. *BCL2* was downregulated by HER3 knockdown and *BAX* was also downregulated, indicating that HER3 inhibition might not change eventual apoptotic status since two genes which have opposite functions on apoptosis are decreased (Supplementary Fig. S4).

### Knockdown of HER3 inhibits anchorage-independent growth of AM cells

Anchorage-independent growth is one of the hallmarks of cancer cells, which is associated with the tumorigenic and metastatic potential of cancer cells and is also considered a marker for in vitro transformation [36]. Besides, the NRG1/HER3/HER2 axis is reported to trigger anchorage-independent growth of basal-like/triple-negative breast cancer cells [37]. Here, to examine the effect of HER3 knockdown on anchorage-independent growth, AM cells were transfected with control or HER3 siRNA and cultured in semisolid agar medium that supports the formation of colonies of transformed cells. The control siRNA-transfected cells successfully grew in semisolid culture and formed colonies (Fig. 3G, H, upper panels), whereas the number and size of colonies were decreased for HER3 siRNA-transfected cells (Fig. 3G, H, lower panels). MTT assay confirmed the significant reduction of colonies for HER3-inhibited cells compared with that for control cells (43.3 ± 10.6% and 25.0 ± 7.78% reductions in SM2-1 and MMG-1 cells, respectively) (Fig. 3G, H). These results imply the involvement of HER3 in regulating the anchorage-independent growth of AM cells.

### HER3 reactivation by NRG1 restores impaired anchorage-independent growth in HER3-knockdown AM cells

**Fig. 4:**
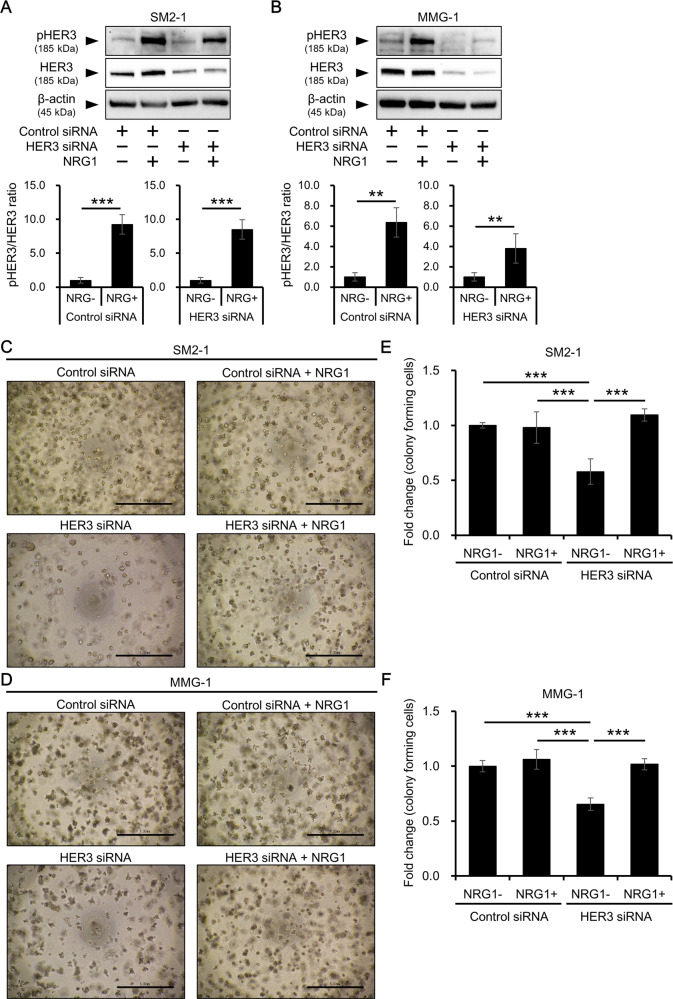
Effects of HER3 reactivation by NRG1 on the anchorage-independent growth of AM cells. AM cells were transfected with control or HER3 siRNA in the presence of vehicle control or NRG1 (10 ng/ml) and evaluated for colony formation/anchorage-independent growth. Status of HER3 phosphorylation in (**A**) SM2-1 and (**B**) MMG-1 cells was determined. β-actin was also measured as a loading control. Experiments were independently repeated three times. Representative blot images (upper) and mean ± SD of pHER3/HER3 ratio (lower) are shown. Original uncropped blot images are shown in Supplementary Fig. S5. **p < 0.01 and ***p < 0.001. Representative images of (**C**) SM2-1 and (**D**) MMG-1 cells cultured in semisolid agar layers. Scale bars = 1.0 mm. **E**, **F** The colonies formed in the agar layers were collected and quantified by MTT assay. Experiments were independently repeated three times and mean ± SD of fold change of absorbance relative to the control siRNA-transfected condition in (**E**) SM2-1 and (**F**) MMG-1 cells is shown. ***p < 0.001.

To confirm the involvement of HER3 in the regulation of anchorage-independent growth of AM cells, HER3 was reactivated by NRG1 in HER3-downregulated AM cells. HER3 was shown not to be phosphorylated without stimulation and NRG1, one of the ligands for HER3, significantly induced the phosphorylation of HER3 in control siRNA-transfected AM cells (Fig. 4A, B and Supplementary Fig. S5). When the HER3 siRNA-transfected cells were further incubated with NRG1, phospho-HER3 was significantly induced in both cell lines (Fig. 4A, B and Supplementary Fig. S5). Then, the anchorage-independent growth was evaluated using cells transfected with control or HER3 siRNA in the presence or absence of NRG1. Formation of colonies was significantly reduced for HER3 siRNA-transfected cells compared with that for control siRNA-treated cells (42.1 ± 11.6% and 34.6 ± 5.69% reductions in SM2-1 and MMG-1 cells, respectively), and the activation of HER3 by NGR1 restored the formation of colonies (Fig. 4C, D). MTT assay confirmed that the reduced colony formation in HER3-inhibited cells was recovered by the treatment with NRG1 (Fig. 4E, F).

### YAP is involved in the function of HER3 to regulate anchorage-independent growth of AM cells

**Fig. 5:**
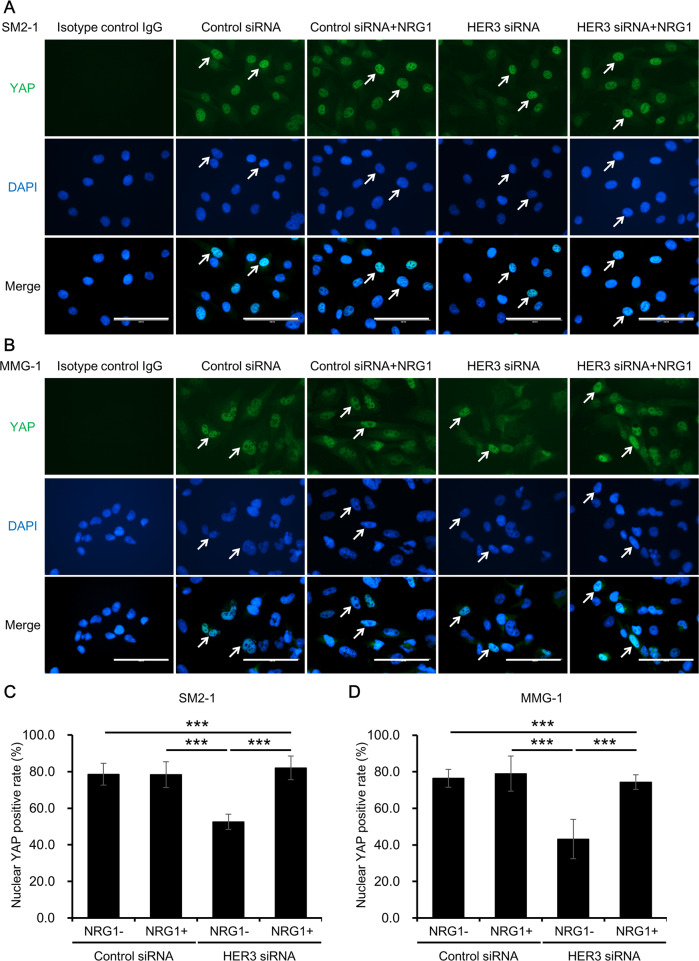
Effect of HER3 knockdown and reactivation on the localization of YAP. AM cells were transfected with control or HER3 siRNA in the presence of vehicle control or NRG1 (10 ng/ml) and evaluated for YAP nuclear translocation. Experiments were independently repeated three times and representative immunocytochemical images of (**A**) SM2-1 and (**B**) MMG-1 cells are shown. Arrows indicate representative cells with nuclear YAP. Scale bars = 100 μm. Mean ± SD of nuclear YAP-positive rate in (**C**) SM2-1 and (**D**) MMG-1 cells. Experiments were independently repeated three times and three images were captured from different fields of each well. Nuclear YAP-positive rate was calculated by dividing the number of nuclear YAP-positive cells by the total number of DAPI-positive cells from the three different fields. ***p < 0.001.

To gain further insights regarding signals underlying HER3 function, the status of signaling molecules was analyzed. YAP is a transcriptional co-activator and effector of the Hippo pathway, and is reported to construct regulatory pathways with NRG1 and HER3 and/or HER4 [38, 39]. Activation of the NRG1/HER3/YAP axis causes nuclear translocation of YAP, which further activates the transcription of downstream genes and promotes anchorage-independent growth [38]. Next, the nuclear translocation of YAP was assessed to investigate the involvement of YAP in the action of HER3 and anchorage-independent growth (Fig. 5). YAP mainly localizes in the nucleus in a steady state, while the inhibition of HER3 decreased the nuclear YAP. Reactivation of HER3 by NRG1 induced the nuclear translocation of YAP in HER3-inhibited AM cells (Fig. 5A, B). The rate of positivity for nuclear YAP was further calculated from the immunocytochemistry images (Fig. 5C, D). Inhibition of HER3 significantly decreased the rate of positivity for nuclear YAP and treatment with NRG1 significantly restored it in HER3-inhibited AM cells (Fig. 5C, D). Nuclear localization of YAP was also confirmed by western blotting. In accordance with immunostaining, inhibition of HER3 significantly decreased nuclear YAP and treatment with NRG1 restored it in HER3-inhibited AM cells (Supplementary Figs. S6A and S7). There was no significant difference of cytoplasmic YAP among conditions (Supplementary Figs. S6B and S7). The expression of downstream genes of YAP, i.e., connective tissue growth factor (*CTGF*) and cysteine-rich angiogenic inducer 61 (*CYR61*) [40, 41], were assessed to confirm the activation of NRG1/HER3/YAP axis. The gene expressions of *CTGF* and *CYR61* were significantly downregulated by HER3 knockdown and additional treatments with NRG1 restored the expressions of *CTGF* and *CYR61* (Supplementary Fig. S6C, D), indicating the activation of YAP signaling through NRG1 and HER3.

We also analyzed other signaling molecules that are reported to regulate anchorage-independent growth of various tumor cells [42]: Akt (downstream of PI3K), proto-oncogene tyrosine-protein kinase Src (Src), extracellular signal-regulated kinase (ERK), c-Jun N-terminal kinase (JNK), and p38 mitogen-activated protein kinase (p38 MAPK). No significant changes were observed in the phosphorylation status of these signaling molecules in AM cells (Supplementary Figs. S8–10).

### AM cells are sensitive to deruxtecan, the cytotoxic part of a HER3-targeted ADC

Considering that HER3 affects anchorage-independent growth of AM cells, it should be beneficial to target and inhibit HER3 in order to prevent tumor progression. Recently, a HER3-targeted ADC, patritumab deruxtecan, has been developed [32, 33], which may have potential value for AM patients positive for HER3. Since patritumab deruxtecan is not commercially available, the effect of its cytotoxic part deruxtecan, on cell viability of AM cells was assessed to evaluate sensitivity of AM cells to this drug. Deruxtecan inhibits DNA topoisomerase I and prevents cell proliferation. The test concentration range of deruxtecan was determined based on the serum concentration of deruxtecan in patients treated with HER3-targeted ADC [43, 44]. The viability of deruxtecan-treated cells differed among the cell types (Supplementary Fig. S11). Notably, in SM2-1 and MMG-1 cells, the viability was significantly decreased even at the lowest concentration (0.3125 nM) compared with that under dimethyl sulfoxide (DMSO)-treated conditions (Supplementary Fig. S11). Besides, the IC_50_ of deruxtecan was significantly low for AM cells compared with that for NHEM, with values of 1.00 ± 0.291, 0.642 ± 0.236, and 3.07 ± 1.07 nM for SM2-1, MMG-1, and NHEM, respectively. The effect of deruxtecan on anchorage-independent growth of AM cells was also assessed. Deruxtecan obviously decreased formation of colonies even at the lowest concentration (0.3125 nM) in AM cells (Supplementary Fig. S12A). MTT assay confirmed that deruxtecan significantly decreased the anchorage-independent growth (Supplementary Fig. S12B).

### Activation of YAP signal by NRG1 does not affect drug resistance of AM cells

YAP is reported to be involved in drug resistance of cancer cells. YAP induces the transcription of ABC multi-drug transporters which promote drug efflux [45]. YAP also promotes chemoresistance by blocking the process of autophagy-related cell death [46]. Given that, the effect of YAP activation by NRG1 on the drug resistance of deruxtecan-treated cells was assessed. NRG1 treatment did not cause significant change of cell viability in deruxtecan-treated AM cells, implying that activation of YAP does not affect drug resistance of AM cells to deruxtecan (Supplementary Fig. S13).

## Discussion

AM is a rare type of cutaneous melanoma found in acral locations with a poorer prognosis than other subtypes of cutaneous melanomas [1–7]. In a clinical setting, AM is treated with combined approaches, however, effective treatments for AM have not been established because of its low incidence and unique characteristics [9–11]. Notably, immunotherapy is less effective for AM partly owing to the low tumor mutation burden [47]. With the aim of evaluating the potential of HER3 as a novel therapeutic target for AM, the expression and function of HER3 in AM tissues of patients and in AM cell lines were assessed in this study.

HER3 is reportedly highly expressed in malignant tumors and, since HER3 promotes tumor progression, the correlation of HER3 and patient survival has been identified in several tumors. High expression of HER3 was correlated with shorter survival in tumors such as basal-like breast cancer, gastric adenocarcinoma, and advanced prostate cancer [37, 48, 49]. In AM, HER3 expression was also significantly correlated with worse DFS, as reported in other tumors. However, unlike the case for DFS, MSS was not significantly different between HER3-positive and HER3-negative patients. Generally, MSS is influenced by the systemic treatment of metastatic lesions, whereas DFS reflects the original tumor characteristics more accurately. Besides, HER3 positivity is also associated with Breslow thickness, an important melanoma staging and prognostic factor [50]. In this study the value of Breslow thickness was greater in the HER3-positive group than in the HER3-negative group, indicating that the HER3-positive group has a more advanced stage and poorer prognosis. Considering these results, HER3 expression is likely to be related to the prognosis of AM.

Downregulation of HER3 significantly inhibited anchorage-independent growth of AM cells, although it did not affect cell proliferation in conventional two-dimensional culture. Generally, HER3 is known to promote the proliferation of cancer cells through activating several signals such as PI3K/Akt and MAPK [48, 49, 51]. However, in the current results, cell proliferation and the status of PI3K/Akt and MAPK signaling molecules were not affected by HER3 inhibition. These findings are similar to those observed in a previous study. In that study, activation of the NRG1/HER3/HER2 axis induced anchorage-independent growth of basal-like/triple-negative breast cancer cells, without affecting cell proliferation [37]. Regarding signals involved in the regulation of anchorage-independent growth, YAP, an effector of the Hippo pathway, reportedly forms autocrine loops with HER3 and regulates the anchorage-independent growth of ovarian cancer cells [38]. When HER3 is inhibited, Hippo kinase cascade phosphorylates YAP and phospho-YAP is degraded. This causes a reduction of nuclear YAP and suppresses downstream gene expression, which in turn inhibits anchorage-independent growth. Consistent with these reports, HER3 stimulation with NRG1 restored the impaired anchorage-independent growth of HER3-inhibited AM cells. Under these conditions, nuclear localization of YAP was also restored by NRG1 treatment in HER3-inhibited AM cells. Besides, YAP downstream genes *CTGF* and *CYR61* were downregulated by HER3 knockdown and additional treatments with NRG1 significantly restored it. *CTGF* and *CYR61* are both known to enhance anchorage-independent growth of cancer cells [40, 41], thus these YAP downstream genes might be involved in the action of NRG1/HER3/YAP axis to regulate anchorage-independent growth of AM cells. Thus, it is likely that the NRG1/HER3/YAP axis plays a role in regulating anchorage-independent growth and that this axis might contribute to tumor progression.

Furthermore, Src and ERK are reported to act as upstream of YAP. In lung adenocarcinoma cells, PTPL1 knockdown induced the activation of Src/ERK signal and eventually promoted YAP nuclear translocation and activation [52]. In skin, Src directly targets YAP at the sites of Y341/357/394 which affect transformation of normal keratinocytes [53]. Although the changes of pSrc and pERK did not reach the significant difference in our experimental setting, slight increase of pSrc and pERK was observed in some biological replicates and there is a possibility that these signals may act as upstream of YAP and affect oncogenic function. On the other hand, the status of Akt, Src, ERK, JNK, and p38 MAPK was not changed by HER3 inhibition although these signaling molecules are known to regulate growth of cancer cells [48, 49, 51]. In addition to these signaling molecules, anchorage-independent growth is reported to be regulated by various signals such as integrin/FAK/SFKs, Ras, Rho, or Hippo signaling pathways [42]. Furthermore, microRNA (miR)-143 and miR-450, which target HER3, are reported to regulate anchorage-independent growth [54–56]. Thus, these molecules may also have potentials to be involved in the regulation of anchorage-independent growth, although further investigation is required.

In this research we used two AM cell lines and found that the cell lines gave slightly different responses to each treatment although the tendencies were the same. It might partly be caused by the differences of the characteristics of cells, as AM cell lines have various features such as source of tissue (primary/metastasis) and genomics (genomic mutations/amplifications) [57, 58]. Although it is preferable to perform experiments with other AM cell lines to strengthen the findings, our results are based on limited number of cell lines because AM cell lines are rare and usually not in commercial. We note that this limitation needs to be addressed in the future research.

We understand the importance of performing in vitro analyses and analyses with a xenograft mouse model using HER3-targeted ADC, to further confirm the effect and utility of HER3-targeted ADC as a novel treatment for AM. However, at present, poor accessibility to ADC for research purposes impedes experiments with the entire ADC. Thus, to indirectly demonstrate the utility of HER3-targeted ADC, the expression of HER3 and sensitivity of AM cells to deruxtecan were assessed. NHEM was less sensitive to deruxtecan compared to AM cells, which might be caused by the highly proliferative ability of tumor cells. In addition, sensitivity to deruxtecan was different between SM2-1 and MMG-1 cell lines. Because these cell lines have different characteristics [57, 58], which might affect the proliferative ability and eventual sensitivity to deruxtecan. Even though the differences of IC_50_ between NHEM and AM cells were small, HER3-targeted ADC is expected to have more chances to bind to AM cells because HER3 is significantly highly expressed in AM cells compared to NHEM. Thus, we believe that our results still support translational relevance of HER3-targeted therapy for AM treatment. In future research projects, we will consider performing experiments with HER3-targeted ADC both in vitro and in vivo by generating our own HER3-targeted ADC.

In conclusion, we found that HER3 was expressed in AM tissues and its expression was correlated with patient survival. HER3 was also shown to be expressed in AM cell lines and to regulate anchorage-independent growth partly through affecting the NRG1/HER3/YAP axis. HER3 may serve as a promising therapeutic target for AM and the use of HER3-targted ADC might be a potential therapeutic approach for AM.

## Materials and methods

### Patients

A retrospective study on AM was performed in accordance with the guidelines of the Declaration of Helsinki. Immunohistochemical analysis of HER3 in AM tissues of patients was approved by the Ethics Committee of Kyushu University Hospital (Approval no. 30-363, approved on November 27, 2018). Written informed consent was obtained from patients before their inclusion in the study. A total of 72 AM patients who had been treated at the Department of Dermatology of Kyushu University Hospital (Fukuoka, Japan) between January 2009 and March 2019 were included. The diagnosis was confirmed by at least three experienced dermatopathologists. Clinical and demographic information on the patients was acquired from their files.

### Immunohistochemistry

Formalin-fixed, paraffin-embedded AM tissues in the archives of Kyushu University Hospital were used. Tissues were cut into 4-μm-thick sections and stained via slightly modified versions of previously reported methods [59, 60]. These sections were incubated with primary antibody (rabbit anti-human HER3/ErbB3, 1:250, 12708S; Cell Signaling Technology, Danvers, MA) for 30 min at room temperature and further incubated with secondary antibody (N-Histofine Simple Stain AP MULTI, 414261; Nichirei Biosciences, Tokyo, Japan) for 30 min at room temperature. Next, the sections were treated with FastRed II (415261; Nichirei Biosciences), a chromogenic substrate, and counterstained with hematoxylin (30002; Muto Pure Chemicals, Tokyo, Japan). Stained sections were observed and images were captured using a Nikon ECLIPSE 80i microscope (Nikon, Tokyo, Japan).

### Evaluation of HER3 immunohistochemical staining

HER3 expression in AM tissues was evaluated using a semi-quantitative method based on the H-score [61]. The staining intensity of HER3 was classified as follows: 0: no staining, 1+: weakly positive, 2+: moderately positive, and 3+: strongly positive. The H-score was obtained by multiplying the staining intensity (0–3+) by the percentage of HER3-positive cells. Two dermatologists (K.T. and T.I.), who were blinded to the patients’ clinical information, evaluated HER3 expression independently.

### Reagents

Deruxtecan (CS-004512; ChemScene, Deerpark, NJ) was dissolved in DMSO (07-4860-5; Sigma-Aldrich, St. Louis, MO) and used at a final concentration of 0.3125–10 nM. NRG1 (100-03; PeproTech, London, UK) was dissolved in distilled water (10977015; Thermo Fisher Scientific, Waltham, MA) and used at a final concentration of 10 ng/ml. DMSO or distilled water (final concentration for both 0.1%) was used as a vehicle control.

### Cell culture

NHEM (KM-4109; Kurabo Industries, Osaka, Japan) were cultured in DermaLife Ma Comp Kit (LMC-LL0039; Kurabo Industries). The AM cell lines SM2-1 (kindly provided by Dr. Hiroshi Murata, Nagano Municipal Hospital, Nagano, Japan) and MMG-1 (kindly provided by Dr. Akifumi Yamamoto, the former Professor, Saitama Medical University International Medical Center, Hidaka, Japan) were used. Both cell lines were established from male, Japanese AM patients. SM2-1 was derived from AM tissue of in-transit metastasis, whereas MMG-1 was derived from primary skin lesion. Genetically, SM2-1 bears *BRAF V600E* and *PTEN C136Y* mutation but lack *KIT* expression, and MMG-1 bears *BRAF V600E* and expresses *KIT* [57, 58]. Cells were cultured in RPMI-1640 (R8758; Sigma-Aldrich) supplemented with 10% fetal bovine serum (174012; Nichirei Biosciences) and 100 U/ml penicillin-streptomycin (15140122; Thermo Fisher Scientific). Cells were passaged by trypsinization at 80% confluence and the medium was changed every 2 days. Cells were confirmed to be mycoplasma-free using CycleavePCR Mycoplasma Detection Kit (CY232; Takara Bio Inc, Kusatsu, Japan). Cell morphology was observed using a microscope and images were obtained by a microscope camera system (Nikon).

### Immunocytochemistry

Cells were seeded in eight-well μ-Slides (80826, 10,000 cells/well; ibidi GmbH, Martinsried, Germany) and incubated for 72 h at 37 °C in 5% CO_2_. Cells were stained for HER3 following a slightly modified version of a previously reported method [62]. After fixation and blocking, cells were incubated with isotype control (1:200, 910801; BioLegend, San Diego, CA) or rabbit anti-human HER3/ErbB3 (1:200) at 4 °C overnight. Cells were further incubated with a secondary antibody (goat anti-rabbit IgG Alexa Fluor^®^488, 1:400, A11008; Thermo Fisher Scientific) for 30 min at room temperature, washed, and covered with Vectashield mounting medium containing DAPI (H-1200; Vector Laboratories, Burlingame, CA). HER3 expression was observed using an EVOS FL fluorescence microscope (Thermo Fisher Scientific). The intensity of HER3 fluorescence was measured using ImageJ software (National Institutes of Health, Bethesda, MD) from three different fields of three wells.

### siRNA transfection

To knock down HER3 in AM cells, either negative control siRNA (AM4611; Invitrogen, Carlsbad, CA) or HER3 siRNA (s4780; Invitrogen) was transfected using Lipofectamine RNAiMAX (13778; Thermo Fisher Scientific), in accordance with the manufacturer’s instructions. Cells were seeded in 6-well plates (2.0 × 10^5^ cells/well), 12-well plates (1.2 × 10^5^ cells/well), or 96-well plates (3000 cells/well) and incubated for 24 h at 37 °C in 5% CO_2_. Cells were then transfected with the siRNAs (final concentration of 10 nM) and incubated for 1–5 days at 37 °C in 5% CO_2_. The efficiency of HER3 knockdown was assessed by quantitative reverse-transcription polymerase chain reaction (qRT-PCR) and western blotting.

### Cell proliferation assay

The number of viable cells among siRNA-transfected AM cells was assessed using Cell Counting Kit-8 (CCK-8, 343-07623; Dojindo, Kumamoto, Japan). Cells were seeded in 96-well plates (3000 cells/well) and incubated for 24 h at 37 °C in 5% CO_2_. Cells were then transfected with control or HER3 siRNA and incubated for 1–5 days at 37 °C in 5% CO_2_. Next, CCK-8 solution was added to each well, followed by incubation for 2 h at 37 °C. The absorbance at 450 nm was measured by a microplate reader (Bio-Rad Laboratories, Hercules, CA).

### Toxicity assay and IC_50_ determination

To investigate the toxicity and IC_50_ of deruxtecan, cells were seeded in 96-well plates (3000 cells/well), incubated for 24 h at 37 °C in 5% CO_2_, and treated with medium containing DMSO (0.1%) or various concentrations of deruxtecan (range 0.3125–10 nM). The deruxtecan concentration was determined based on the serum deruxtecan levels of patients (~10 nM) [43, 44]. At 48 h of incubation, CCK-8 solution was added, followed by incubation at 37 °C in 5% CO_2_. After 2 h, absorbance at 450 nm was measured using a microplate reader (Bio-Rad Laboratories). IC_50_ was calculated using nonlinear regression analysis in GraphPad Prism 7 software (GraphPad Software, San Diego, CA). To evaluate effects of NRG1 on drug resistance of AM cells, cells were treated with medium containing DMSO (0.1%) or various concentrations of deruxtecan (range 0.3125–10 nM) with vehicle control or NRG1 (10 ng/ml). At 48 h of incubation, viable cells were quantified with CCK-8 solution as mentioned above.

### qRT-PCR

Total RNA was extracted using the RNeasy Mini Kit (74104; Qiagen, Hilden, Germany) following the manufacturer’s instructions. RNA was reverse-transcribed to cDNA using a PrimeScript RT Reagent Kit (RR037; Takara Bio Inc) and used for qPCR with TB Green Premix Ex Taq (RR420; Takara Bio Inc). The PCR conditions were 95 °C for 30 s and 40 cycles of 95 °C for 5 s and 60 °C for 20 s. The expression level of each gene was normalized using the cycle threshold (Ct) of β-actin (*ACTB*) as an internal control, and relative gene expression compared with that under control conditions was calculated using the comparative Ct method. The sequences of the primers used are listed in Supplementary Table S2.

### Western blotting

Protein was extracted from cells using M-PER mammalian protein extraction reagent (78503; Thermo Fisher Scientific) supplemented with protease inhibitor cocktail (P8340; Sigma-Aldrich). Protein samples were mixed with sample buffer (30566-22; Nacalai Tesque, Kyoto, Japan) and heated at 96 °C for 5 min. Western blotting was subsequently performed as previously reported [62]. When needed, membranes were cut based on the molecular weight to separately hybridize with different antibodies. The primary antibodies were summarized in Supplementary Table S3. The secondary antibody was goat anti-rabbit IgG horseradish peroxidase (1:10,000, 7074; Cell Signaling Technology). The resulting bands were detected using SuperSignal West Pico Chemiluminescent Substrate (34580; Thermo Fisher Scientific) and the ChemiDoc XRS Plus System (Bio-Rad Laboratories). The intensity of the bands was quantified using ImageJ software.

### Colony formation assay (anchorage-independent growth)

Cells were transfected with control or HER3 siRNA with vehicle control or NRG1 (10 ng/ml) and incubated for 24 h at 37 °C in 5% CO_2_. Colony formation was evaluated using CytoSelect^TM^ 96-Well Cell Transformation Assay (CBA-135; Cell Biolabs, San Diego, CA), in accordance with the manufacturer’s instructions. The siRNA-transfected cells were harvested by trypsinization, mixed with 10×CytoSelect Agar Solution and CytoSelect Matrix Diluent, and added to the wells containing a base agar layer (2500 cells/well). Seeded cells were covered with culture medium (50 μl/well) and incubated at 37 °C in 5% CO_2_ for 7 days. The agar layers were then solubilized with Matrix Solubilization Solution (125 μl/well), transferred to a new 96-well plate (100 μl/well), mixed with MTT solution (10 μl/well), and incubated at 37 °C in 5% CO_2_ for 2 h. After the incubation, detergent solution (100 μl/well) was added and the plate was further incubated for 2 h at room temperature in the dark with gentle shaking. The absorbance of the resultant products at 570 nm was measured by a microplate reader (Bio-Rad Laboratories).

### Effect of deruxtecan on anchorage-independent growth of AM cell lines

AM cells were seeded in semi-solid agar layer as described above and further incubated with media containing DMSO (0.1%) or various concentrations of deruxtecan (range 0.3125–10 nM) at 37 °C in 5% CO_2_ for 7 days. The colony forming cells were quantified by MTT assay as described above.

### Evaluation of YAP nuclear translocation

To evaluate the localization of YAP, cells were transfected with control or HER3 siRNA and treated with vehicle control or NRG1 (10 ng/ml). Treated cells were fixed with 4% paraformaldehyde (163-20145; Fujifilm Wako Pure Chemical, Osaka, Japan) for 15 min at room temperature. After washing with Dulbecco’s phosphate-buffered saline (DPBS, 293-72601; Fujifilm Wako Pure Chemical), cells were treated with 0.1% Triton X-100 (30-5140-5; Sigma-Aldrich) for 15 min and further incubated with 5% bovine serum albumin (A-2153; Sigma-Aldrich) for 30 min at room temperature. Cells were then incubated with isotype control (1:100) or anti-YAP antibody (1:100, 14074; Cell Signaling Technology) at 4 °C for overnight. After three washes with DPBS, cells were incubated with goat anti-rabbit IgG Alexa Fluor^®^488 for 60 min at room temperature. After three washes, cells were covered with Vectashield mounting medium containing DAPI. Nuclear localization of YAP was observed under an EVOS FL fluorescence microscope. The proportion of cells positive for nuclear YAP was calculated by dividing the number of nuclear YAP-positive cells by the number of DAPI-positive cells.

### Nuclear/cytoplasmic protein extraction for western blotting

Nuclear/cytoplasmic protein was separately extracted using NE-PER nuclear and cytoplasmic extraction reagents (78833; Thermo Fisher Scientific) following the manufacturer’s instructions. Briefly, cell pellet was suspended with ice-cold CER I, incubated on ice for 10 min, mixed with CER II, centrifuged at 16,000 × *g* for 5 min at 4 °C, and supernatant was then separated as cytoplasmic protein. The remaining pellet was suspended with ice-cold NER and incubated on ice for 40 min while vortex 15 s every 10 min. After the incubation, samples were centrifuged at 16,000 × *g* for 5 min at 4 °C and supernatant was then separated as nuclear protein.

### Statistical analysis

Experiments were repeated at least three times independently to test statistical significance. Results are displayed as the mean ± standard deviation (SD). GraphPad Prism 7 was used to perform statistical analyses. The normality of the data distribution was examined using the Shapiro–Wilk test. The significance of differences between two groups was examined by Student’s unpaired two-tailed *t* tests. However, when the data deviated from a normal distribution, Mann–Whitney U test was used. To analyze differences among three or more groups, one-way analysis of variance and Dunnett’s multiple comparisons test were used. To assess the correlation of HER3 expression intensity with patients’ clinical factors, *t*-test and Fisher’s exact test were used. The survival of patients was analyzed by the Kaplan–Meier method and the log-rank test. *p* < 0.05 was considered statistically significant.

## Supplementary information

Supplementary Materials

## Data Availability

All data generated or analyzed during this study are included in this article and its Supplementary Information files.
