# Supplementary material for: Human epidermal growth factor receptor 3 serves as a novel therapeutic target for acral melanoma
Source: Cell Death Discov. 2023 Feb 10;9:54. doi: 10.1038/s41420-023-01358-5 (PMC9918519; doi:10.1038/s41420-023-01358-5)
Supplement: Supplementary file 1 — Supplementary Materials [file 41420_2023_1358_MOESM1_ESM.pdf]

Human epidermal growth factor receptor 3 serves as a novel therapeutic target for acral melanoma

Running title: HER3 as a therapeutic target for acral melanoma

Yuka Tanaka[a], Takamichi Ito[a,*], Yumiko Kaku-Ito[a], Keiko Tanegashima[a], Gaku Tsuji[a], Makiko Kido-Nakahara[a], Yoshinao Oda[b], and Takeshi Nakahara[a]

[a]Department of Dermatology, Graduate School of Medical Sciences, Kyushu University, Fukuoka 812-8582, Japan
[b]Department of Anatomic Pathology, Graduate School of Medical Sciences, Kyushu University, Fukuoka 812-8582, Japan

*Corresponding author
Takamichi Ito
Department of Dermatology, Graduate School of Medical Sciences, Kyushu University, 3-1-1 Maidashi, Higashi-ku, Fukuoka 812-8582, Japan
Tel.: +81-92-642-5585, Fax: +81-92-642-5600
E-mail: ito.takamichi.657@m.kyushu-u.ac.jp

This file contains:
Supplementary Figures S1-S13
Supplementary Tables S1-S3

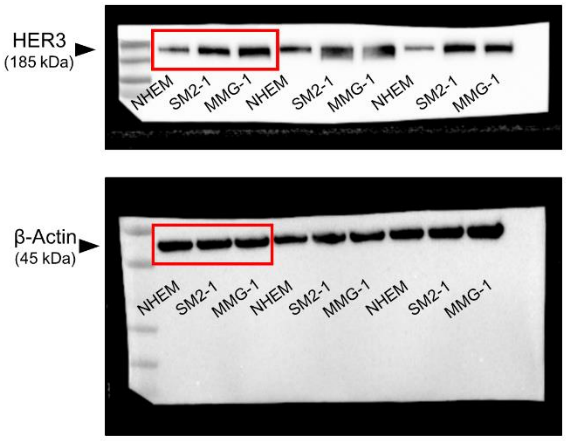

Supplementary Figure S1. Original full length western blot images presented in Fig. 2B. HER3 and β-actin protein expression in NHEM, SM2-1, and MMG-1 cells was determined by western blotting. Unedited original images of blots are shown. The samples were derived from three independent experiments. The red boxes indicate the cropped areas shown in Fig. 2B.

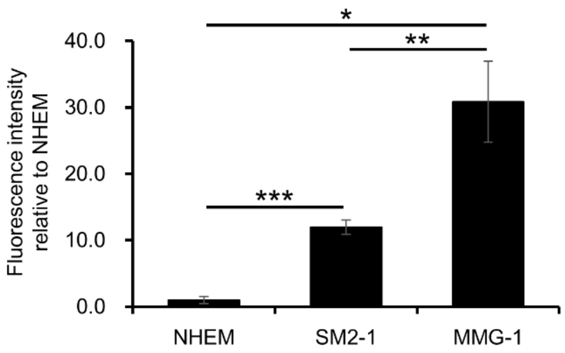

Supplementary Figure S2. Fluorescence intensity of HER3 in NHEM, SM2-1, and MMG-1 cells. HER3 was stained by immunocytochemistry (Fig. 2C) and its fluoresecence signal was quantitated using ImageJ software. Signals were measured from three different fields of three wells. *p < 0.05, **p < 0.01, and ***p < 0.001.

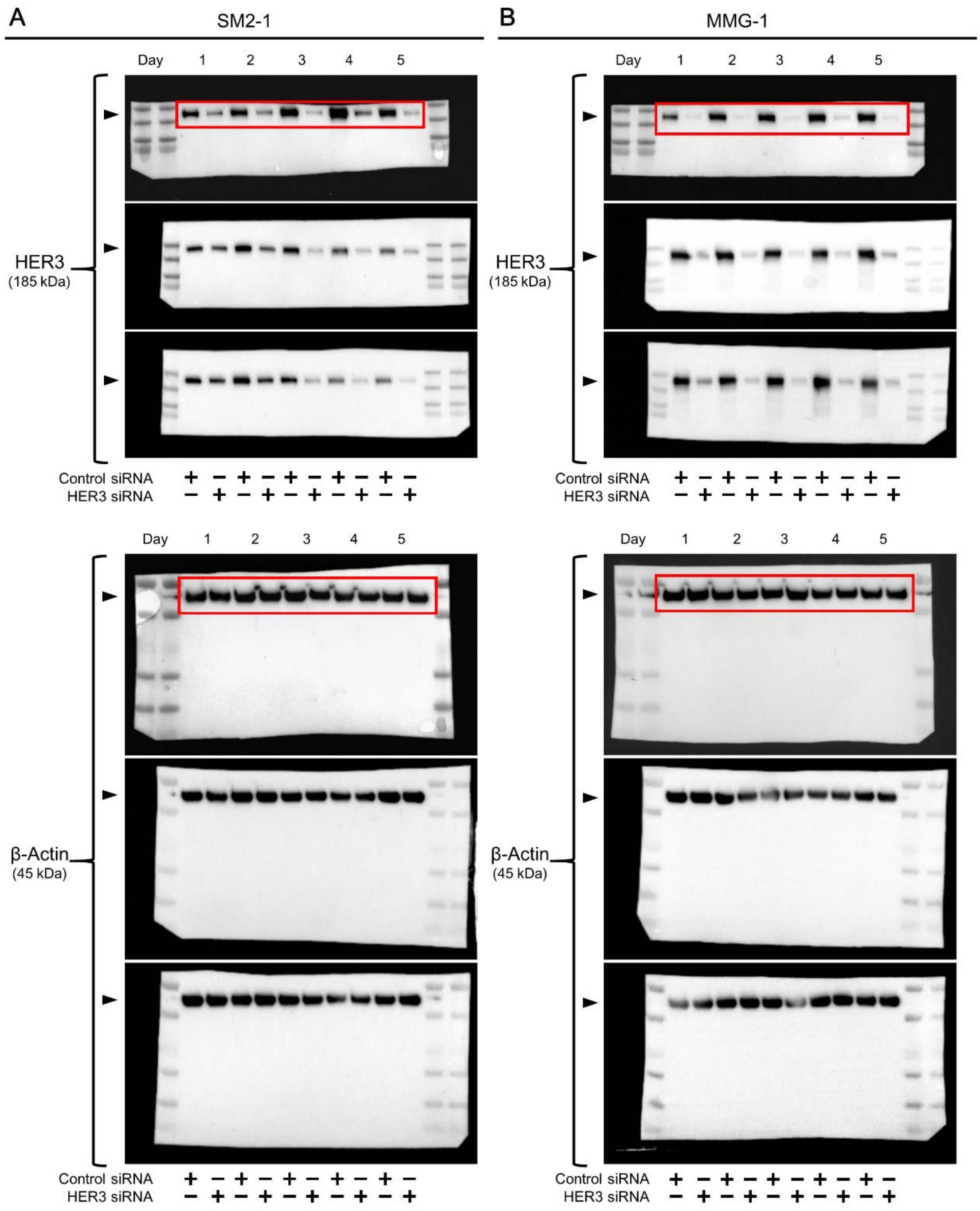

Supplementary Figure S3. Original full length western blot images presented in Fig. 3C and D. HER3 and β-actin protein expression in control or HER3 siRNA-transfected (**A**) SM2-1 and (**B**) MMG-1cells was determined by western blotting. Unedited original images of blots are shown. The samples were derived from three independent experiments. The red boxes indicate the cropped areas shown in Fig. 3C and D.

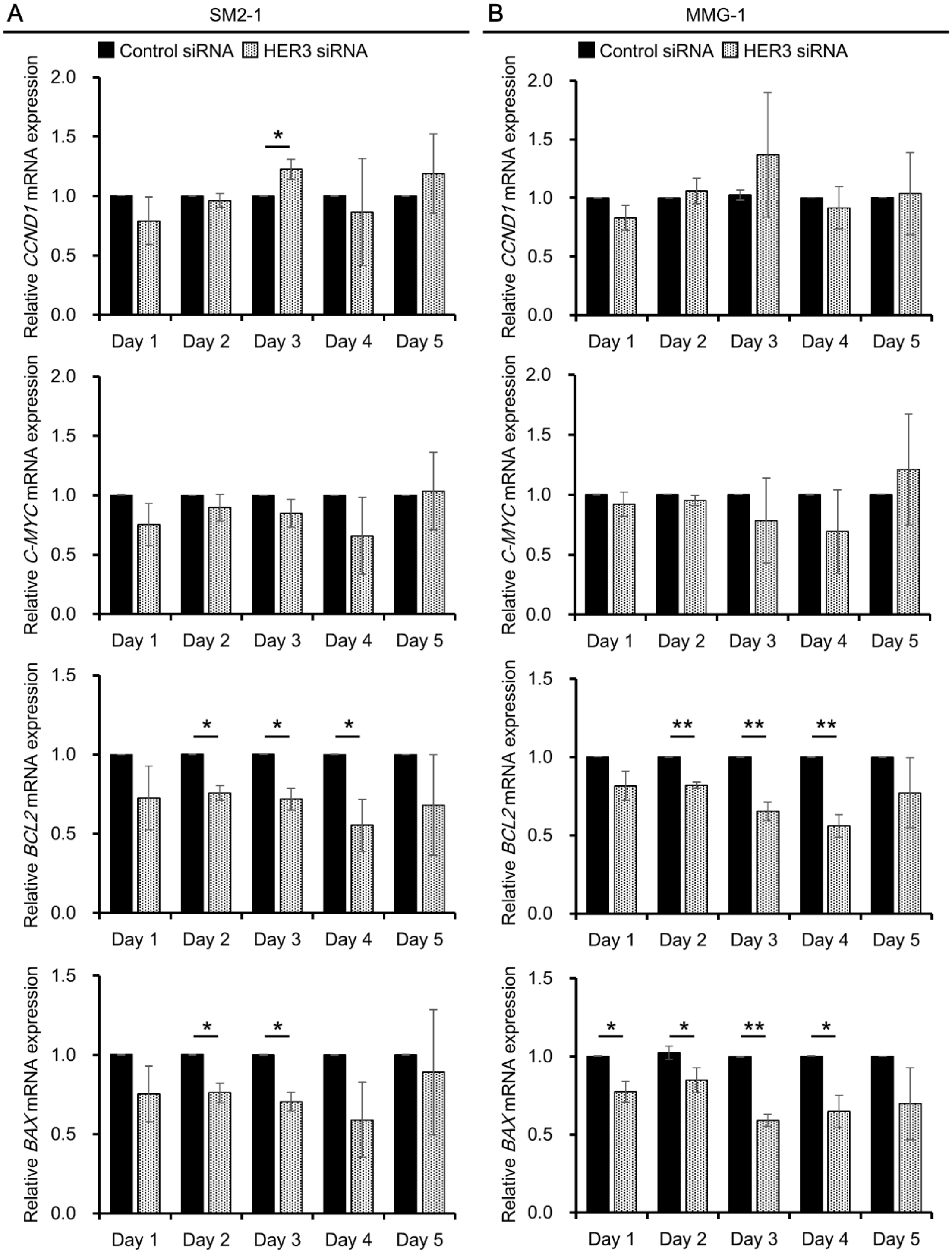

Supplementary Figure S4. Expression of *CCND1, C-MYC, BCL2,* and *BAX* in siRNA-transfected AM cells. Gene expression of *CCND1*, *C-MYC, BCL-2*, and *BAX* was measured in control or HER3 siRNA-transfected (**A**) SM2-1 and (**B**) MMG-1 cells. Data are the mean ± SD of three independent experiments. *$p < 0.05$ and **$p < 0.01$.

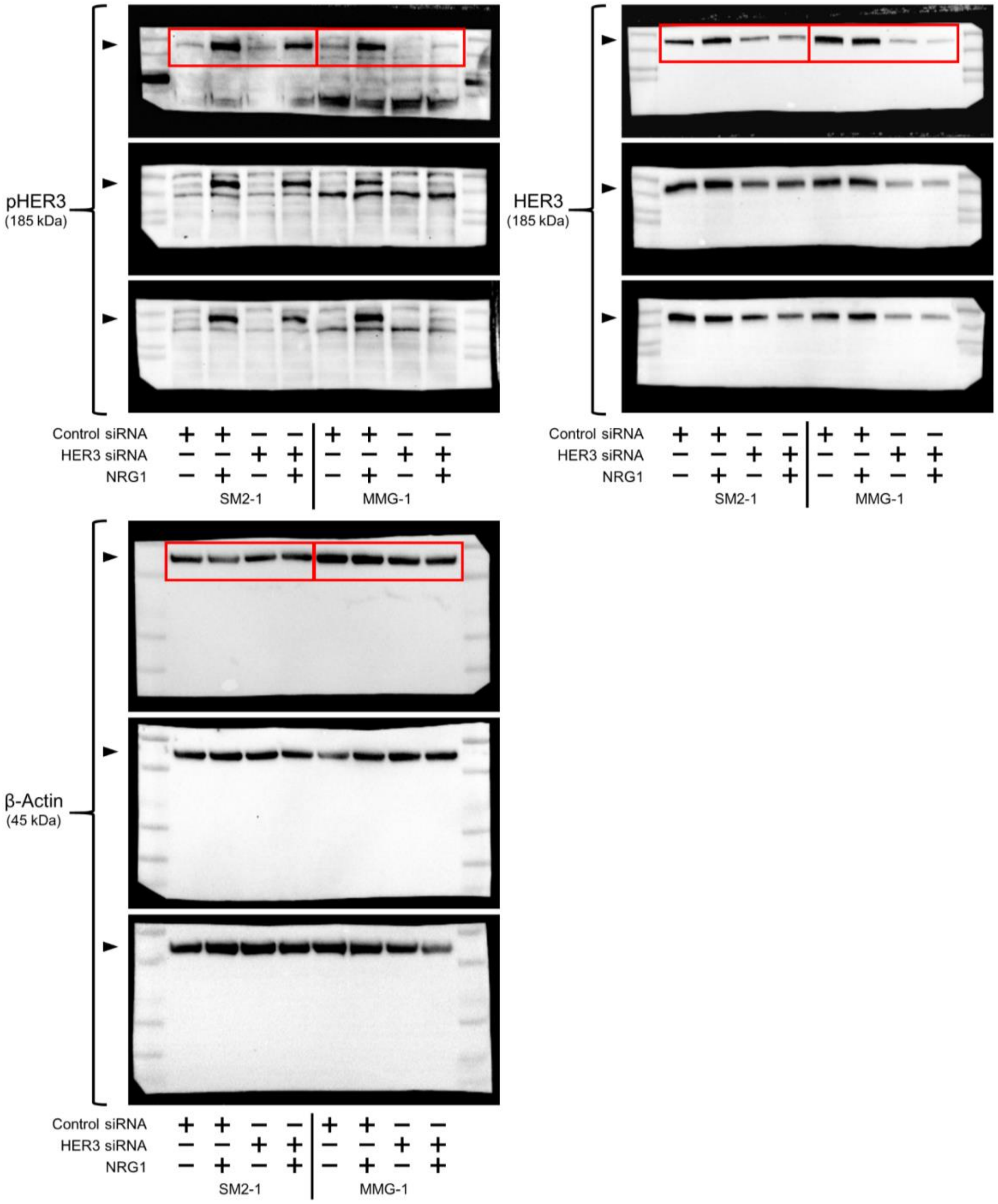

Supplementary Figure S5. Original full length western blot images presented in Fig. 4A and B. Phospho-HER3 (pHER3) and HER3 protein expression in control or HER3 siRNA-transfected SM2-1 and MMG-1 cells treated with vehicle control or NRG1 (10 ng/mL) was determined by western blotting. β-actin was also measured as a loading control. Unedited original images of blots are shown. The samples were derived from three independent experiments. The red boxes indicate the cropped areas shown in Fig. 4A and B.

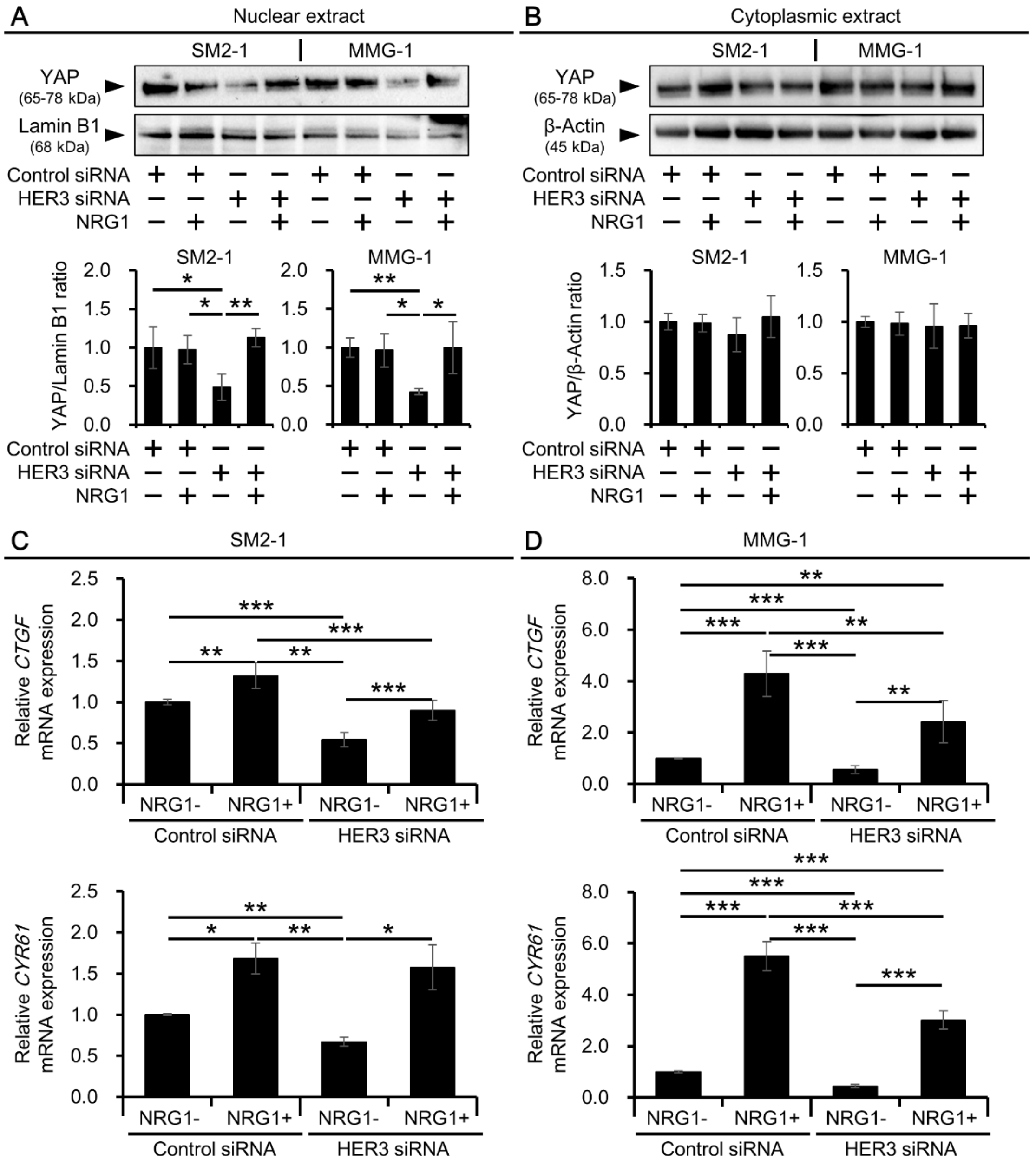

Supplementary Figure S6. Effects of HER3 knockdown and NRG1 treatment on YAP signaling. AM cells were transfected with control or HER3 siRNA with vehicle control or NRG1 (10 ng/mL) for 48 h and evaluated for YAP localization and downstream gene expressions. (**A** and **B**) Protein expression of YAP in (**A**) nuclear and (**B**) cytoplasmic fraction of AM cells. Lamin B1 or β-actin were used as internal controls of nuclear or cytoplasmic extract, respectively. Representative blot images (upper) and YAP protein expressions calculated from three independent experiments (lower) are shown. Full-length blot images are shown in Supplementary Fig. S7. (**C** and **D**) The expressions of representative YAP downstream genes *CTGF* and *CYR61* in (**C**) SM2-1 and in (**D**) MMG-1 cells were evaluated. Data are the mean ± SD of three independent experiments. *$p < 0.05$, **$p < 0.01$, and ***$p < 0.001$.

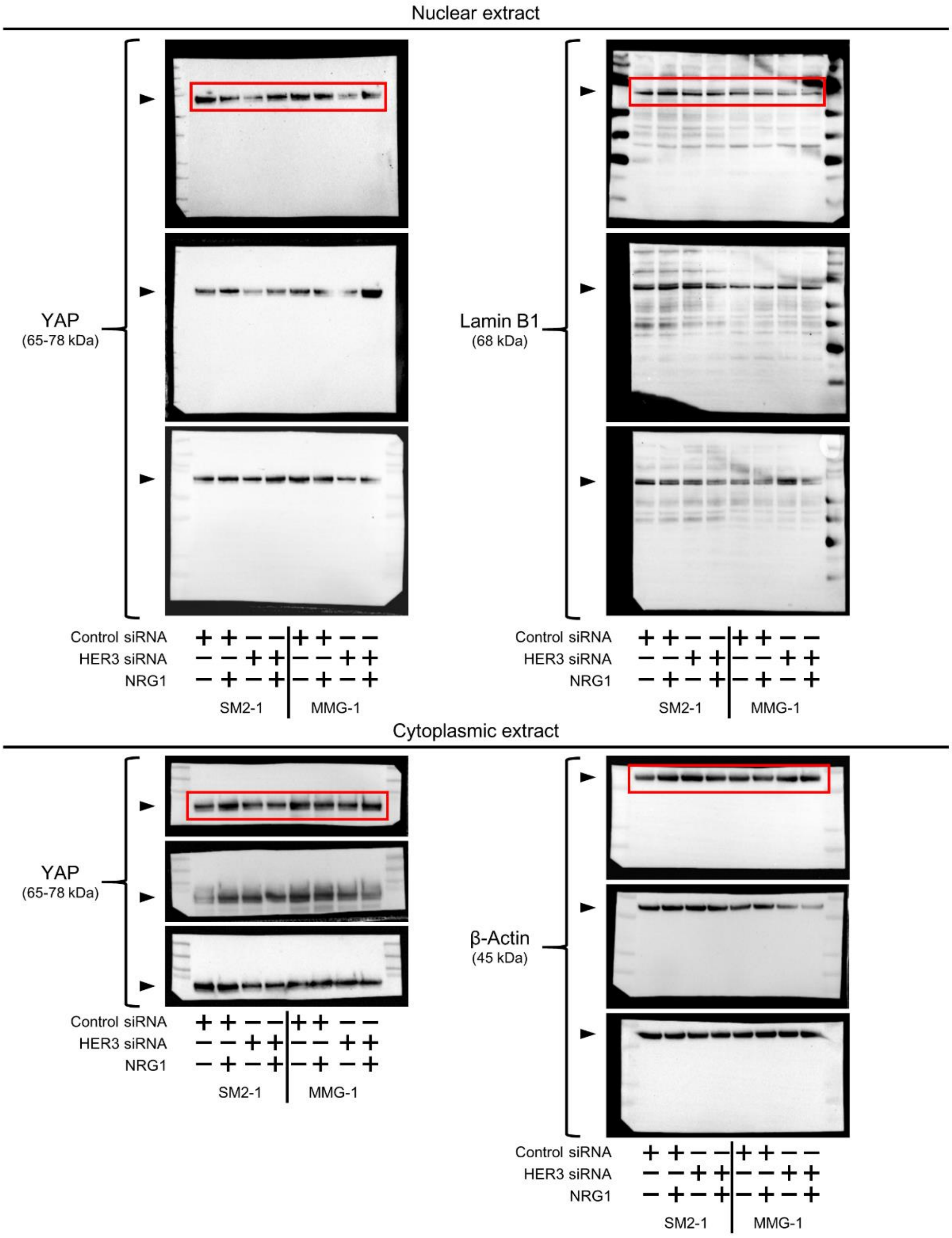

Supplementary Figure S7. Original full length western blot images presented in Supplementary Fig. S6A and B. Protein expression of YAP, Lamin B1, and β-actin in control or HER3 siRNA-transfected AM cells treated with vehicle control or NRG1 (10 ng/mL) was detected by western blotting. Nuclear and cytoplasmic protein was separetely extracted. Unedited original images of blots are shown. The samples were derived from three independent experiments. The red boxes indicate the cropped areas shown in Supplementary Fig. S6A and B.

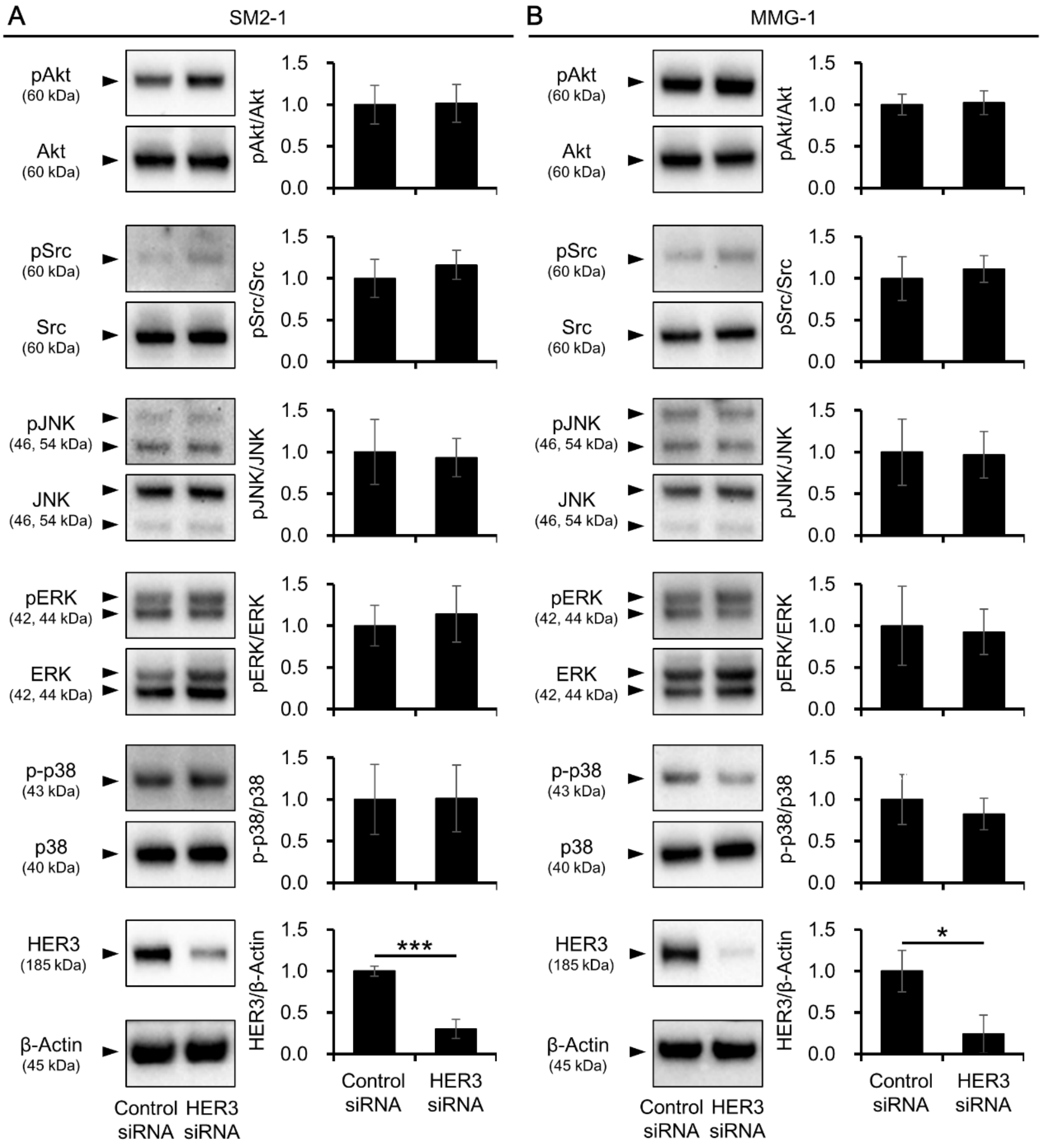

Supplementary Figure S8. Phosphorylation status of signaling molecules in siRNA-transfected AM cells. Protein expression of signaling molecules and their phosphorylated forms in control or HER3 siRNA-transfected (**A**) SM2-1 and (**B**) MMG-1 cells was detected by western blotting. β-actin was measured as a loading control. HER3 was also measued to confirm knockdown of HER3 by siRNA. The signal of each molecule was analyzed using ImageJ software. The samples were derived from three independent experiments. Full-length blot images are shown in Supplementary Fig. S9 and S10. * $p < 0.05$ and ***$p < 0.001$.

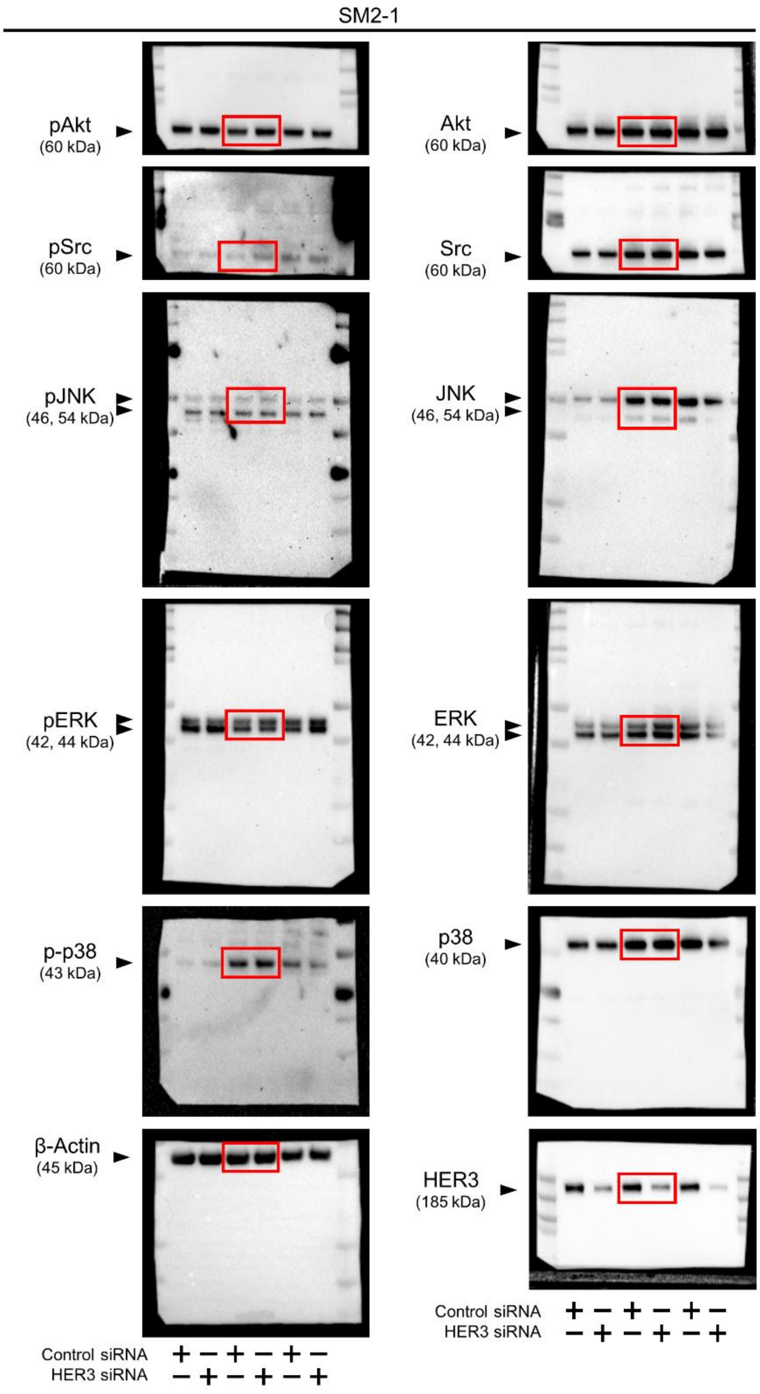

Supplementary Figure S9. Original full length western blot images presented in Supplementary Fig. S8A. Protein expression of signaling molecules and their phosphorylated forms, β-actin, and HER3 in control or HER3 siRNA-transfected SM2-1 cells was detected by western blotting. Unedited original images of blots are shown. The samples were derived from three independent experiments. The red boxes indicate the cropped areas shown in Supplementary Fig. S8A.

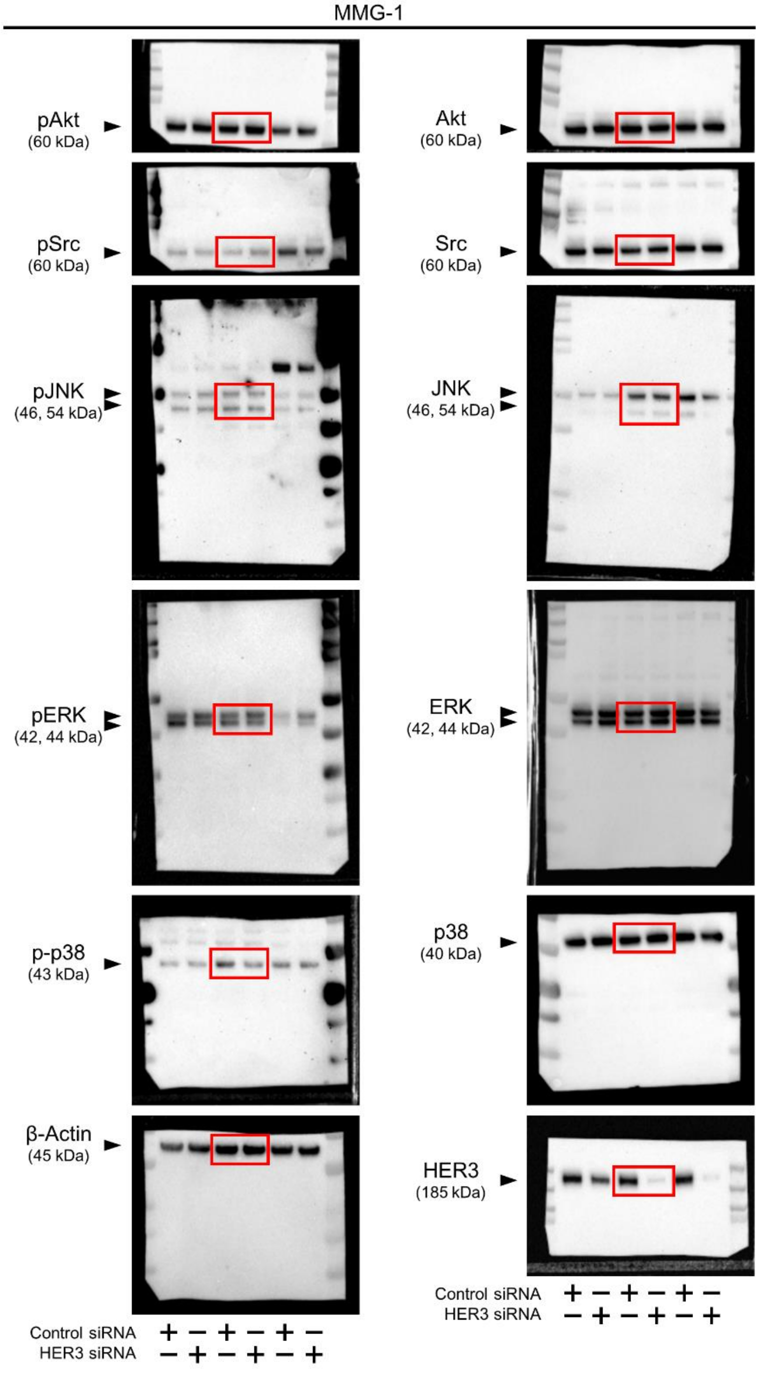

Supplementary Figure S10. Original full length western blot images presented in Supplementary Fig. S8B. Protein expression of signaling molecules and their phosphorylated forms, β-actin, and HER3 in control or HER3 siRNA-transfected MMG-1 cells was detected by western blotting. Unedited original images of blots are shown. The samples were derived from three independent experiments. The red boxes indicate the cropped areas shown in Supplementary Fig. S8B.

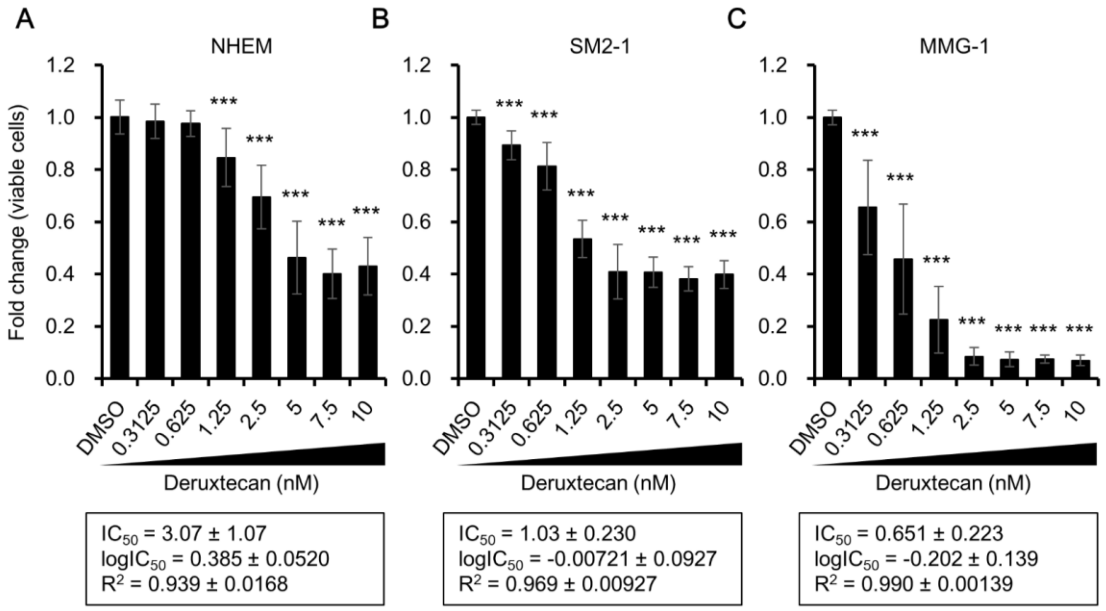

Supplementary Figure S11. Toxicity and IC_{50} of deruxtecan in normal and malignant melanocytes. Toxicity of deruxtecan, the payload of a HER3-targeted ADC, was assessed. Viability of (**A**) NHEM, (**B**) SM2-1, and (**C**) MMG-1 cells treated with DMSO (0.1%) or various concentrations of deruxtecan (0.3125–10 nM) for 48 h is shown. Cell viability was measured using a formazan-based method. Mean ± SD of cell viability is shown as a bar chart (upper) and the values of IC_{50} are indicated in the boxes under the bar chart. Treatments were performed in five wells for each concentration and experiments were independently repeated three times. ****p* < 0.001.

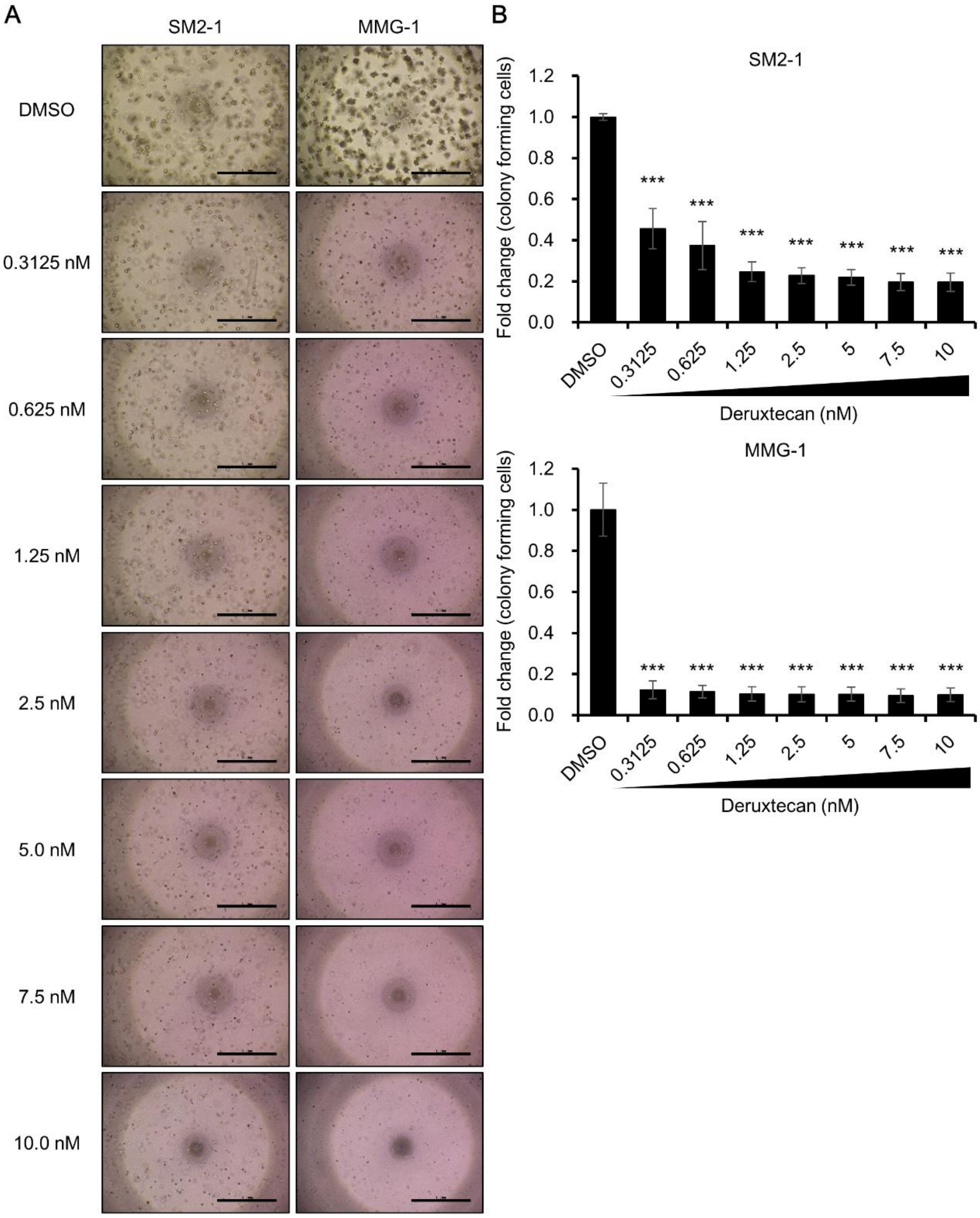

Supplementary Figure S12. Effects of deruxtecan on anchorage-independent growth of AM cells. Effect of deruxtecan on anchorage-independent growth was assessed using a semi-solid culture system. (**A**) Representative images of SM2-1 and MMG-1 AM cells treated with DMSO (0.1%) or various concentrations of deruxtecan (0.3125–10 nM) for 7 days. Scale bars =1.0 mm. (**B**) Colony forming cells were quantitated by MTT assay. Experiments were performed in two wells for each concentration and mean ± SD of fold change of absorbance at 570 nm obtained from three independently repeated experiments is shown. ***p < 0.001.

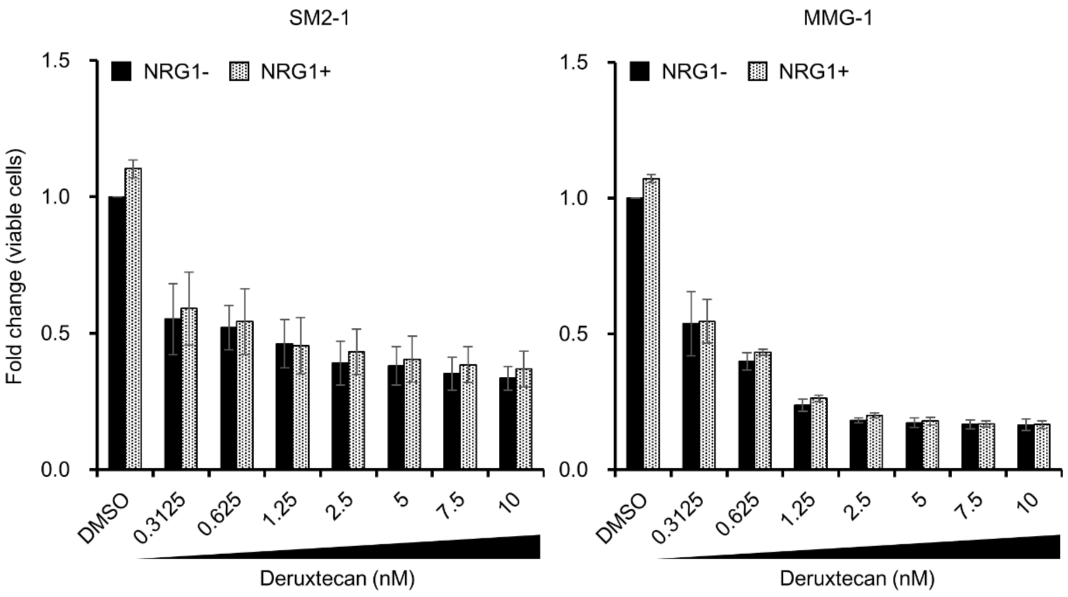

Supplementary Figure S13. Effects of YAP activation by NRG1 on drug resistance of AM cells. To evaluate the effects of YAP activation on drug resistance of AM cells against deruxtecan, cells were treated with medium containing DMSO (0.1%) or various concentrations of deruxtecan (range 0.3125–10 nM) with vehicle control or NRG1 (10 ng/mL). At 48 h of incubation, viable cells were quantified using a formazan-based method. Mean ± SD of cell viability is shown. Treatments were performed in five wells for each concentration and experiments were independently repeated three times.

Supplementary Table S1. HER3 positivity and clinicopathological factors

	Positive (n = 23)	Negative (n = 49)	*p*-value
Age (year, mean ± SD)	66.7 ± 16.1	64.8 ± 15.8	0.649
Sex (n)			
Male	11	23	>0.999
Female	12	26	
Tumor site (n)			
Hand	4	6	0.463
Foot	12	33	
Nail apparatus	7	10	
Breslow thickness (mm, mean ± SD)	4.26 ± 3.25	2.38 ± 3.42	0.029
AJCC stage			
I, II	13	39	0.052
III, IV	10	10	

AJCC, American Joint Committee on Cancer; SD, standard deviation

Supplementary Table S2. Primer sequences for qRT-PCR

Gene symbol	Primer sequence
ACTB	Sense: 5′-ATTGCCGACAGGATGCAGA-3′
	Antisense: 5′-GAGTACTTGCGCTCAGGAGGA-3′
BAX	Sense: 5′-GGACGAACTGGACAGTAACATGG-3′
	Antisense: 5′-GCAAAGTAGAAAAGGGCGACAAC-3′
BCL2	Sense: 5′-ATCGCCCTGTGGATGACTGAG-3′
	Antisense: 5′-CAGCCAGGAGAAATCAAACAGAGG-3′
CCND1	Sense: 5′-GCTGCGAAGTGGAAACCATC-3′
	Antisense: 5′-CCTCCTTCTGCACACATTTGAA-3′
C-MYC	Sense: 5′-GAGGCGAACACACAACGTCTT-3′
	Antisense: 5′-CGCAACAAGTCCTCTTCAGAAA-3′
CTGF	Sense: 5′-CTTGCGAAGCTGACCTGGAAGA-3′
	Antisense: 5′-CCGTCGGTACATACTCCACAGA-3′
CYR61	Sense: 5′-GGAAAAGGCAGCTCACTGAAGC-3′
	Antisense: 5′-GGAGATACCAGTTCCACAGGTC-3′
HER3	Sense: 5′-CTATGAGGCGATACTTGGAACGG-3′
	Antisense: 5′-GCACAGTTCCAAAGACACCCGA-3′

Supplementary Table S3. Primary antibodies used for western blotting

Antibody	Host	Manufacturer	Catalog no.	Dilution
Anti-human β-actin	Rabbit	Cell Signaling Technologies	#4970	1:2,000
Anti-Akt	Rabbit	Cell Signaling Technologies	#9272	1:1,000
Anti-pAkt	Rabbit	Cell Signaling Technologies	#4969	1:2,000
Anti-ERK	Rabbit	Cell Signaling Technologies	#9102	1:1,000
Anti-pERK	Rabbit	Cell Signaling Technologies	#4370	1:2,000
Anti-HER3/ErbB3	Rabbit	Cell Signaling Technologies	#12708	1:1,000
Anti-pHER3(Tyr1197)	Rabbit	Cell Signaling Technologies	#4561	1:1,000
Anti-JNK	Rabbit	Cell Signaling Technologies	#9258	1:1,000
Anti-pJNK	Rabbit	Cell Signaling Technologies	#4668	1:1,000
Anti-Lamin B1	Rabbit	Cell Signaling Technologies	#12586	1:1,000
Anti-p38 MAPK	Rabbit	Cell Signaling Technologies	#8690	1:1,000
Anti-p-p38 MAPK	Rabbit	Cell Signaling Technologies	#4511	1:1,000
Anti-Src	Rabbit	Cell Signaling Technologies	#2123	1:1,000
Anti-pSrc	Rabbit	Cell Signaling Technologies	#2101	1:1,000
Anti-YAP	Rabbit	Cell Signaling Technologies	#14074	1:1,000
